# Implementation Determinants of Zimbabwe National Occupational Safety and Health Policy in Willowvale Industrial Area, Zimbabwe

**DOI:** 10.3390/ijerph19031424

**Published:** 2022-01-27

**Authors:** Tarisayi Mkungunugwa, Patrick Opiyo Owili, Adamson Sinjani Muula, Hsien-Wen Kuo

**Affiliations:** 1 School of Postgraduate Studies, Adventist University of Africa, Nairobi 00100, Kenya; mkungunugwam@aua.ac.ke; 2 Research Capacity Strengthening Division, African Population and Health Research Center, Nairobi 00100, Kenya; 3 College of Medicine, University of Malawi, Blantyre 312225, Malawi; amuula@medcol.mw; 4 Institute of Environmental and Occupational Health Sciences, School of Medicine, National Yang Ming Chiao Tung University, Taipei 112, Taiwan

**Keywords:** occupational safety and health, policy, implementation, determinants, Zimbabwe

## Abstract

Zimbabwe introduced the National Occupational Safety and Health Policy (ZNOSHP) in August 2014 with the vision and mission to eliminate occupational accidents, injuries, diseases, and fatalities and to promote Occupational Safety and Health (OSH). This study was therefore aimed at exploring the individual- and organizational-level determinants of ZNOSHP’s implementation. Data were collected from 309 workers in the Willowvale industrial area in Harare, Zimbabwe. Negative binomial regression models were used to explore the determinants of ZNOSHP’s implementation. After adjustment, participant’s knowledge of ZNOSHP (Incidence Rate Ratio, IRR = 1.32; 95% Confidence Interval, CI: 1.19–1.46; *p* ≤ 0.001), production department (IRR = 1.13; 95% CI: 1.03–1.26; *p* ≤ 0.05), company years of operation (IRR = 1.33; 95% CI: 1.21–1.46; *p* ≤ 0.001), participants who identified several implementation barriers (IRR = 1.12; 95% CI: 1.01–1.25; *p* ≤ 0.001), and agricultural industry were associated with higher rates of ZNOSHP’s implementation. In conclusion, individual- and organizational-level determinants of implementation of OSH standards were explored, and positive associations were found. Policy implementation, enforcement, and follow up strategies need to be developed in order to ensure adherence to safety measures. This study should be extended to other parts of Zimbabwe in order to develop evidence-based policy.

## 1. Introduction

Occupational safety and health (OSH) policies exists to guarantee the health, safety, and wellbeing of all workers [1,2,3]. However, these are often overlooked in Zimbabwe, which has led to 20,641 serious injuries among workers and over 400 deaths from injuries in the years between 2009 and 2013 [4]. The most common occupational accidents and diseases are recorded and classified by the International Labor Office [5]. Most of these accidents and diseases are a result of the machinery used, the work, external environments, and other worker-associated factors [6,7,8,9,10,11], and these have been exacerbated by employers’ slow adoption and implementation of the OSH management system [4]. Consequently, the slow implementation of the OSH management system may finally lead to a long-term negative effect on the cost and productivity of businesses, workers, and society.

Additionally, the industrial production capacity in Zimbabwe increased from 10% in 2009 to 57% in 2011, and it is also expected that occupational accidents, injuries, diseases, and deaths will increase. Hence, this led to the introduction of the Zimbabwe National Occupational Safety and Health Policy (ZNOSHP) in August 2014 with the vision and mission to eliminate occupational accidents, injuries, diseases, and fatalities and to promote OSH in Zimbabwe [4]. The introduction of this policy enshrined the constitutionality and the rights of employees to have labor practices that are fair and safe, and subsequently, an efficient work process, quality products, and loss reduction resulting from accidents, injuries, diseases, and deaths at work. Even though ZNOSHP focuses on self-regulation by employers, it also provides for employees’ rights to fair and safe labor practices, to be trained on OSH risks and their effects, to be involved in the development of OSH risk mitigation strategies, and to decline any work that has been rendered unsafe [4].

The national body responsible for the planning, development, and implementation of OSH-related programs in Zimbabwe is the National Social Security Authority (NSSA), which recommended that all industries should be responsible for promoting OSH, employment of OSH professionals, appointment of competent persons for machine operability, establishment of OSH committees, and adoption of OSH management systems [4]. However, both the employers’ and employees’ attitudes remain important towards the implementation of the safety and health standards and the reduction in occupational accidents and diseases, which are always shaped by several individual and organizational determinants involving human factors (i.e., behavior or action of workers), worksite factors (i.e., poor working conditions), management factors (i.e., lack of management’s vision and support on safety issues), industry sector (i.e., hard labor jobs), and external factors (i.e., legal framework and enforcement) [12,13,14,15].

To date, only about 16% of the companies in Zimbabwe have adopted and implemented the policy document, with some citing barriers such as inadequate resources, and lack of ZNOSHP’s knowledge and commitment from the management [16]. However, these factors that have contributed to the lack of implementation of OSH policy or the low response have not been adequately explored in Zimbabwe. This study therefore aimed to determine the individual- and organizational-level of ZNOSHP implementation and to identify the barriers that contributed to the low implementation rates, before exploring the determinants of implementation in the Willowvale industrial area in Harare, Zimbabwe.

## 2. Materials and Methods

### 2.1. Study Design and Participants

A cross-sectional study design was conducted in various industries located in the Willowvale industrial area in Harare with a total population of 989 employees. In the postwar era, the industrial zone was commonly associated with the working class southern European immigrants, but currently it is known for black Zimbabweans and the descendants of Zambian, Mozambiquean, and Malawian immigrants [17]. Several manufacturing industries dealing with products such as electronics, textiles, vehicles, and food production are found in this area. It is also popularly known for being the world’s largest tobacco auction center [17]. The participants were therefore drawn from different industrial firms including agricultural, manufacturing, commerce and distribution, building, construction, and mining firms. After a study sample was calculated with a margin of error of 5%, a confidence level of 95%, and a response distribution of 50%, a sample size of 203 was required, which was distributed proportionately by sector. However, the participants in the companies showed a lot interest in this study, and 309 individuals voluntarily participated. The high response rate, however, did not affect the representation from different companies. Instead, it increased the statistical power. A structured self-administered questionnaire was then administered for data collection from the employees who were willing to participate in the study. Both the outcome and the independent variables were collected. This study was ethically approved by the Research Ethics Committee of the University of Eastern Africa, Baraton, and we also obtained another administrative approval from the NSSA’s Research and Development Department.

### 2.2. Data Collection

Enumerators were selected and trained on the questionnaire items before data collection. In the process of data collection, the enumerators were required to explain to the respondents the details of the questionnaire before requesting the participants who were willing to participate in the study to sign an informed consent form. The participants were not coerced to participate and had the right to withdraw from the study at will.

As the participants were responding to the questionnaire items in a room, enumerators were available to assist in case the participants needed further clarifications on the questionnaire items. Even though the questionnaire was self-administered, the enumerators would also check after completion to ensure that all the questionnaire items were answered so as to avoid missing data. Nonetheless, the presence of the enumerator did not influence the response rate. Participation in the study was voluntary. Supervision also took place in order to maintain and ensure quality in the data collection. Data were collected in the month of March 2021.

Once data were collected, they were then entered into MS Excel before they were imported into the statistical software for final analysis. The data were anonymized to protect the privacy of the respondents. Moreover, data were secured using a password that was only known to the researchers in order to protect information leakage.

### 2.3. Outcome Variable

The outcome variable ‘implementation of ZNOSHP’ had 33 indicators that were classified as use of personal protective equipment (PPE i.e., helmet, face mask, googles, earplugs, gloves, apron, and safety boots), sanitation requirements (i.e., adequate ventilation, enough lighting, female and male toilets, waste bins, and water availability including for drinking), emergency preparedness (i.e., electric and explosive signs, slippery floor signs, steps signs, falling objects signs, fire warning alarms, emergency exit signs, readily accessible first-aid kit, waiting/resting rooms available, and changing rooms available), and other safety components in the ZNOSHP (i.e., employed safety and health professional, regular safety inspections by NSSA, safety policy being implemented, work safety training at employment, in-service work safety training, workers’ compensation insurance, emergency preparedness and response plan, reporting of accidents to the nearest inspector, calculation of lost time due to injury frequency, maintained accident and lost time register, availability of the accident register to NSSA inspectors, and study of hazard and operability of new equipment).

### 2.4. Independent Variables

The independent variables considered as the determinants comprised both the individual-level variables as well as the organization-level variables. The individual-level indicators included: ‘knowledge about ZNOSHP’, which was measured using three indicators (i.e., awareness, training, and availability of ZNOSHP document) and categorized as low (≤1 indicator), moderate (2 indicators), and high (all the 3 indicators); gender (i.e., male vs. female); age group (i.e., ≤29 years, 30–39 years, and ≥40 years); educational level (i.e., secondary and below vs. college/university); employment position was categorized as senior position (i.e., manager and supervisor) and junior position (i.e., skilled and unskilled laborers); years of service (i.e., ≤5 years, 6–10 years, and >10 years); terms of contract (i.e., permanent vs. contract); and work shift (i.e., day vs. night). On the other hand, the organization-level variables included: department categorized as administration, production, and other (i.e., sales, purchasing, stores, security, etc.); industry sector (i.e., agricultural, building/construction/mining, commerce/distribution, and manufacturing); company years of operation (i.e., ≤20 years vs. >20 years); and the number of employees (i.e., ≤25 employees vs. >25 employees).

We also explored if the employees’ attitude, depicted in the recommendations, affected the implementation of ZNOSHP. This was measured using the following six binary indicators: regular training to gain skills and safety knowledge, involving workers in the implementation process, empowering safety and health officers, penalties for non-compliance, familiarizing with the key policy requirements, and raising awareness on ZNOSHP by policy makers. The ‘recommendations of the employees’ were then combined and categorized into two groups (i.e., ≤5 indicators = some recommendations; otherwise, all 6 indicators).

Finally, the ‘implementation barriers’ had seven indicators (i.e., lack of skills and knowledge, lack of management commitment and vision, inadequate knowledge on policy benefits, high implementation cost, disempowered safety and health professionals, lack of penalties for non-implementation, and unrealistic safety policy). In the analysis, the barriers were combined and categorized into three group (i.e., ≤2 = low barriers; 3–4 barriers = moderate; and ≥5 barriers = high barriers). The barriers are presumed to cause low levels of policy implementation.

### 2.5. Statistical Analysis

Data were analyzed in three stages. In the first stage of the analysis (descriptive), numbers and proportions of the knowledge, implementation, recommendations, and barriers to implementation of ZNOSHP were presented before combining the key indicators of the dependent variable in subsequent phase of the analysis.

In the second phase of the analysis, the 33 indicators of the dependent variable ‘implementation of ZNOSHP’ were then combined to identify the number of OSH items that the participants indicated as implemented, and hence the count data. The count data were then grouped into two categories (i.e., <20 items vs. ≥20 items) in order to analyze the differences between the categories of the independent variables. A chi-square test was then employed to test the differences between categories of each independent variable by the outcome variable.

In the final stage of our analysis (i.e., inferential), negative binomial regression technique was used to explore the determinants of ZNOSHP’s implementation regression upon the count data, ‘implementation of ZNOSH’ in Willowvale industrial area. The incidence rate ratios (IRRs) and 95% confidence intervals (CI) were then reported for further interpretation. Data were analyzed using STATA version 13.1 [18].

## 3. Results

### 3.1. Descriptive Statistics

#### 3.1.1. Knowledge, Implementation, Recommendations, and Barriers

The sample size in this study was 203 participants. However, most of the employees had expressed their willingness to participate in the study, and hence data were collected from 309—that is, a 152.2% response rate.

Table 1 shows the participants responses on the knowledge and implementation of the ZNOSHP, employees’ recommendations, and implementation barriers. Over a half of the participants indicated that they were aware of the system approach to OSH management (57.7%). Even though the majority of the participants had indicated that they were aware of ZNOSHP (88.8%), and that the OSH policy document was available in their organization (53.0%), few indicated that they had been trained on ZNOSHP (46.7%).

On the individual-level implementation of the ZNOSHP, only 70.5% indicated that they were using safety boots as PPE. The usage of the remaining PPEs (i.e., helmet, face mask, googles, earplugs, gloves, and apron) were very low among the participants, ranging between 23.5% and 38.6%. The majority also indicated that almost all of the sanitation requirements were implemented, which include adequate ventilation (79.0%), toilets for both genders (92.8%), waste bins (94.7%), and water (95.9%) at the organization-level. A few also indicated that the buildings had enough lighting.

Concerning emergency preparedness at the organization-level, over 50% of the participants indicated that there were electric and explosive signs (51.1%), fire warning alarms (81.5%), emergency exit signs (59.9%), and accessible first-aid kits (57.1%). However, few participants stated that slippery floor signs, step signs, falling object signs, resting rooms, and changing room were available. Moreover, the majority indicated that other safety components of ZNOSHP such as regular safety inspections by NSSA (67.1%), conducting in-service work safety training (58.6%), workers’ compensation insurance (55.2%), emergency preparedness, and response plans (52.0%) were available.

Some of the critical safety components of ZNOSHP were not available in the organizations such as the safety and health professional (47.3%), safety policy implementation (40.4%), work safety training at employment (48.8%), accident reporting to the NSSA inspector (36.4%), calculation of lost time due to injury frequency (20.1%), register of accident and lost time (37.0%), and the study of hazard and operability for new equipment (29.8%). Nevertheless, the majority of the employees recommended that there should be regular training to gain skills and safety knowledge, involvement of workers in the implementation process, empowerment of safety and health officers, penalties for non-compliance, familiarization with the key policy requirements, and that policy makers should raise awareness on ZNOSHP.

The barriers associated with the implementation of ZNOSHP were also identified by the participants (Table 1). The majority of employees indicated that there was lack of management’s commitment and vision (59.2%), inadequate knowledge on policy benefits (59.6%), high cost associated with the implementation (64.9%), and disempowered safety and health professionals (50.2%). About a third of the participants also noted lack of work safety skills, knowledge, and penalties for non-implementation as contributing towards non-implementation.

#### 3.1.2. Individual and Organizational Characteristics by ZNOSHP’s Implementation

In the second phase of the analysis, the 33 indicators of “implementation of ZNOSH” were combined to identify the number of OSH items the participants had indicated that were being implemented, and hence the count data. These were then grouped into two categories (i.e., <20 items vs. ≥20 items) in order to analyze the demographic differences by the outcome variable, “implementation of ZNOSH”. Other variables that were combined and categorized included the following concepts: “ZNOSHP knowledge” (i.e., ≤1 indicator = low; 2 indicators = moderate; and all 3 indicators = high), “employees’ implementation recommendations” (i.e., ≤5 indicators = some recommendations; otherwise, all 6 indicators) and “implementation barriers” (i.e., ≤2 = low barriers; 3–4 barriers = moderate; and ≥5 barriers = high barriers).

Table 2 shows the test of differences between the categories of participants by the categorized concept “implementation of ZNOSHP”. The Chi-Square test was used to test the differences between the categories. The participants who indicated a higher rate of implementation had high knowledge of ZNOSHP (55.2%), had more than 10 years of service (43.2%), were working during the night shift (56.5%), were in the production department (36.2%), were in the agricultural sector (60.0%), in a company that had operated for more than 20 years (52.4%), and those that had recommended key implementation issues (31.5%). Additionally, the participants who identified high levels of implementation barriers (51.5%) had higher rates of implementation. These variables were statistically associated with ZNOSHP’s implementation at *p* < 0.05.

### 3.2. Determinants of ZNOSHP Implementation

In the final phase of the analysis, negative binomial regression analysis was used to explore the determinants of ZNOSHP implementation. The unadjusted negative binomial regression models (Table 3) also revealed that those who had high ZNOSHP knowledge, had over 10 years of service, were on a permanent contract, were working during the night shift, were in the production and other departments in a company that had operated for over 20 years and had more than 25 employees, recommended implementation of all requirements, and identified high level of barriers towards implementation were more likely to have a higher rate of occupational safety implementation.

However, after adjusting for all the variables, only ZNOSHP knowledge, department in the organization, industry sector, company years of operation, and those who identified several implementation barriers remained associated with the implementations of the ZNOSHP. Participants who had high levels of ZNOSHP knowledge (IRR = 1.32; 95% CI: 1.19–1.46; *p* ≤ 0.001) were at a higher rate of ZNOSHP implementation than those who had low knowledge levels. Those who were in the production department and other departments respectively had 13% (95% CI: 1.03–1.26; *p* ≤ 0.05) and 11% (95% CI: 0.99–1.25; *p* ≤ 0.10) higher rates of ZNOSHP’s implementation than those in the administration department.

Moreover, those who were in a company that had operated for over 20 years implemented ZNOSHP 1.33 times (95% CI: 1.21–1.46; *p* ≤ 0.001) higher than those who were in companies that had operated for less than 20 years. Moreover, those who identified several implementation barriers (IRR = 1.12; 95% CI: 1.01–1.25; *p* ≤ 0.001) were more likely to implement ZNOSHP than those who noted that there were low implementation barriers. However, those who were in the manufacturing industry had a lower rate of ZNOSHP implementation (IRR = 0.90; 95% CI: 0.80–1.02; *p* ≤ 0.10) than those who were in the industries dealing with agricultural produce.

## 4. Discussion

This study was aimed at determining the individual- and organizational-level of ZNOSHP implementation and identify barriers to implementation of the OSH policy, before exploring the determinants of ZNOSHP implementation in Willowvale industrial area, Harare, Zimbabwe. The results show that 88.8% of the participants were aware of ZNOSHP while only 46.7% indicated that they had been trained on the policy document. The study found that the level of PPE usage (i.e., helmet, face mask, googles, earplugs, gloves, and apron) was very low among the employees. Moreover, companies that had operated for more than 20 years (52.4%) and were engaged with agricultural produce (60.0%) had a higher level of OSH implementation. The negative binomial regression analysis results of the determinants found that employee ZNOSHP knowledge, company’s department, years of operation, industry sector, and barriers were associated with and determined the level of ZNOSHP’s implementation.

The low level of employees trained on ZNOSHP may have contributed to the low usage of PPEs. This, therefore, calls for a training need of employees on OSH requirements in the Willowvale industrial area. Palka and Habek [6] also found that in sectors that have a lot of fatal accidents, the form, efficiency, and method of training contributes majorly to the employee’s awareness of the dangers and preventions techniques, and that the knowledge gained by the employees is dependent on the training quality. The gained knowledge is likely to change employees behavior towards OSH [7]. Other authors have also noted that passive training such as the use of workbooks or on-line learning increases the level of awareness and empowerment [19]. If workers are not knowledgeable on the OSH policy, chances remain that there will be low usage of PPEs, as found in this study, and subsequently lots of accidents that can lead to loss of productive time. In Zimbabwe, some authors have also found that the accidents associated with OSH reduced the employees’ productivity and output [8].

The results also revealed that some of the organizational-level safety components were not implemented in the companies as per the policy requirements, and these included: adequate lighting, slippery floor signs, step signs, falling objects signs, waiting and changing rooms, employing an OSH professional, training on OSH at employment, maintenance of accident register and reporting of accident to NSSA inspectors, calculation of lost time due to injury, and assessing the new equipment for operability and hazard. The non-compliance of the companies may be as a result of some of the barriers that were identified in this study and others.

Apart from the training need, the barriers that were identified by the participants as the main reasons for non-compliance with the OSH policy document in the Willowvale industrial area included lack of commitment and OSH vision on the part of management, inadequate knowledge on policy benefits, high implementation cost, and disempowered safety and health professionals. Loosemore and Andonakis [20] also found that some of the leading barriers to effective implementation of OSH include “implementation costs, language and educational barriers, and a fear of change”. Even though education level was also explored in this study, it was found to be non-significant in the Willowvale industrial area, before and after adjusting for all the other variables. Other authors have also found several other barriers to OSH implementation such as safety awareness, ineffective information and communication, time, cost, production prioritization, inappropriate management behavior, and management of legal compliance, resources, and regulation [9,21,22,23,24].

In this study, however, those who identified many barriers were nonetheless 12% more likely to implement safety standards than those who only identified a few barriers, after adjusting for all the variables. This may be as a result of their knowledge and years of experience with regard to safety issues in the company, as those who had high knowledge of ZNOSHP were 32% more likely to implement safety standards than those who had low knowledge levels, after adjustment. Moreover, before adjusting for all of the variables, those who had worked in the company for more than 10 years were 18% more likely to implement several safety items than their counterparts who had worked for less than 5 years. However, after adjusting for all the variables, the difference attenuated.

Some of the other individual-level indicators that were likely to contribute toward the implementation of ZNOHSP included terms of contract and work shift with those who were on a permanent contract and were working on a night shift having a higher rate of implementation than their colleagues in the reference groups. However, these were only statistically significant before adjustment, and after adjusting for all the variables, the differences attenuated. The results of this study also showed that organization-level variables such as years of operation and industry sector contributed to the implementation, with companies that had been in existence for more than 20 years and were engaged with agricultural produce having a higher level of OSH implementation than their counterparts in other categories. Studies have also suggested that organizational factors such as organizational capacity, industrial sector, and company size are of potential importance [10,11,13,25]. It is therefore expected that companies that have been in existence for many years and are in industries that have a high risk for accidents may have had experience with regards to OSH standards, and hence are more likely to implement an OSH policy than those that have not operated for many years. The reason for companies that were dealing with agricultural produce having a higher rate of safety implementation than those in the manufacturing industry may have been as a result of other requirements in the food industry that insist on the maintenance of high levels of hygienic standards when handling food items. However, this assumption needs to be explored in other studies.

Moreover, the production unit and other department were more likely to implement ZNOSHP than the administration simply because of the differences in the nature of their duties. The administration units are more office- and paperwork-oriented, rather than being hands-on and engaged with the companies’ products.

This study had several strengths and weaknesses. One of the strength of this study was in the exploration of various ZNOSHP concepts that were necessary for safety of employees. Secondly, this study was focused and conducted in the most important industrial area within the country. Several industries and employees in diverse sectors, diverse roles and products are found in the Willowvale industrial area. Thirdly, this study is a foundation towards the assessment and enforcement of a national safety standard in different organizations. Moreover, the advanced analytical technique that was used adds strength to the study. Several variables were controled for in this study. Nevertheless, one of the weakness of this study was in relation to the use of a cross-sectional design, which looks at both the dependent and the independent variables in a snapshot, hence making it difficult to establish a causal relationship. Studies that follow and observe the behavior of the employees are therefore necessary in order to give insightful recommendations, specific to the different organizations involved. Secondly, this study can only be generalized to the Willowvale industrial area and not the entire country. Some other organizations that were not in the study area may be, or may not be, performing well with regard to the implementation of ZNOSHP.

### Policy and Research Implication

Policy implementation and enforcement strategy needs to be clear so that the companies and OSH officers know and understand their responsibilities toward better working conditions and increased productivity in Zimbabwe. The results of this study can therefore inform policy makers to understand that making policies is one thing, while implementating is another. Policy enforcercement agencies should be actively involved in the identification of weak points in the policy implementation, and subsequently develop strategies that will ensure OSH requirements are maintained. This study has encouraged and utilized the policy implementation assessment method from the employees’perspective, and hence agencies should endevour to employ the same. An intervention and a follow-up study is therefore necessary to understand the actual safety measures implemented in various sectors and organizations. This study should also be extended to other parts of the country in order to adequately inform policy actions. Future studies should also consider assessing occupational accidents and disease in different industries. Incentives that would encourage the implementation of OSH policy are also very important and should be considered.

## 5. Conclusions

In conclusion, this study identified the level of ZNOSHP implementation and implementation barriers, and further explored the implementation determinants in the Willowvale industrial area. Several OSH standards were explored and the study found that the use of PPEs was very low among the employees. Moreover, barriers such as management’s commitment and vision, inadequate knowledge on policy benefits, cost associated with implementation, and disempowered OSH professionals were identified. This study also found that, at the individual-level, knowledge on policy matters was a determinant. While, at the organization-level, the company’s department, sector, and years of operation were important determinants of ZNOSHP implementation.

## Figures and Tables

**Table 1:** The descriptive statistics of participant’s response on knowledge, implementation, recommendations, and barriers to implementation of ZNOSHP in Willowvale industrial area, Harare, Zimbabwe (*n* = 309).

Variables	No, *n* (%)	Yes, *n* (%)
**Aware of systems approach to OSH management**	135 (42.3)	184 (57.7)
**Employee ZNOSPH’s knowledge**		
Aware of ZNOSHP	37 (11.6)	282 (88.4)
Trained on ZNOSHP	170 (53.3)	149 (46.7)
ZNOSHP policy document is available	150 (47.0)	169 (53.0)
**Implementation ZNOSHP**		
*Use of personal protective equipment*		
Helmet	214 (67.1)	105 (32.9)
Face mask	198 (62.1)	121 (37.9)
Googles	237 (74.3)	82 (25.7)
Earplugs	226 (70.8)	93 (29.2)
Gloves	196 (61.4)	123 (38.6)
Apron	244 (76.5)	75 (23.5)
Safety boots	94 (29.5)	225 (70.5)
*Sanitation requirements*		
Adequate ventilation	66 (20.7)	252 (79.0)
Enough lighting	180 (56.4)	139 (43.6)
Toilets (Female/Male)	23 (7.2)	296 (92.8)
Waste bins	17 (5.3)	302 (94.7)
Water availability (including drinking)	13 (4.1)	306 (95.9)
*Emergency preparedness*		
Electric and explosive signs	156 (48.9)	163 (51.1)
Slippery floor signs	181 (56.7)	138 (43.3)
Steps signs	237 (74.3)	82 (25.7)
Falling objects signs	209 (65.5)	110 (34.5)
Fire warning alarms	59 (18.5)	260 (81.5)
Emergency exit signs	128 (40.1)	191 (59.9)
Readily accessible first-aid kit	137 (42.9)	182 (57.1)
Waiting/resting rooms available	203 (63.6)	116 (36.4)
Changing rooms available	186 (58.3)	133 (41.7)
*Other safety components in the ZNOSHP*		
Safety & health professional available	168 (52.7)	151 (47.3)
Regular safety inspections by NSSA	105 (32.9)	214 (67.1)
Safety policy being implemented	190 (59.6)	129 (40.4)
Trained on work safety at employment	165 (51.7)	153 (48.0)
Have in-service work safety training	132 (41.4)	187 (58.6)
Have workers’ compensation insurance	143 (44.8)	176 (55.2)
Have emergency preparedness and response plan	153 (48.0)	166 (52.0)
All accidents are reported to the nearest inspector	203 (63.6)	116 (36.4)
Know lost time due to injury frequency calculation	255 (79.9)	64 (20.1)
Organization has a register of accident & lost time	201 (63.0)	118 (37.0)
Accident register available to NSSA inspectors	213 (66.8)	104 (32.6)
Hazard and operability study for new equipment	222 (69.6)	95 (29.8)
**Employees recommendation on ZNOSHP implementation**		
Regular training to gain skills and safety knowledge	8 (2.5)	311 (97.5)
Involving workers in the implementation process	5 (1.6)	314 (98.4)
Empowerment of safety and health officers	3 (0.9)	316 (99.1)
Penalties for non-compliance	37 (11.6)	282 (88.4)
Familiarization with the key policy requirements	2 (0.6)	317 (99.4)
Policy makers should raise awareness on ZNOSHP	3 (0.9)	316 (99.1)
**ZNOSHP implementation barriers**		
Lack of work place safety skills and knowledge	201 (63.0)	118 (37.0)
Lack of management’s commitment and vision	130 (40.8)	189 (59.2)
Inadequate knowledge on policy benefits	129 (40.4)	190 (59.6)
High implementation cost	112 (35.1)	207 (64.9)
Disempowered safety and health professionals	159 (49.8)	160 (50.2)
Lack of penalties for non-implementation	199 (62.4)	120 (37.6)
Unrealistic safety policy	289 (90.6)	30 (9.4)

OSH, Occupational Safety and Health; ZNOSHP, Zimbabwe National Occupational Safety and Health Policy. Bold & Italics: distinguish the variable name from the categories.

**Table 2:** Differences between participant characteristics by implementation of the ZNOSHP in Willowvale industrial area, Harare.

Variables	Total *n* = 309	ZNOSHP Implemented, *n* (%)		*p*-Value ^†^
		<20 Items	≥20 Items	
**Employee ZNOSHP knowledge** ^a^				<0.001
Low	104	87 (83.6)	17 (16.4)	
Moderate	128	100 (78.1)	28 (21.9)	
High	87	39 (44.8)	48 (55.2)	
**Gender**				0.281
Male	223	162 (72.6)	61 (27.4)	
Female	96	64 (66.7)	32 (33.3)	
**Age group**				0.059
≤29	77	55 (71.4)	22 (28.6)	
30–39	160	121 (75.6)	39 (24.4)	
≥40	82	50 (61.0)	32 (39.0)	
**Education level**				0.319
Secondary and below	97	65 (67.0)	32 (33.0)	
College/university	222	161 (72.5)	61 (27.5)	
**Job position** ^b^				0.437
Senior	172	125 (72.7)	47 (27.3)	
Junior	147	101 (68.7)	46 (31.3)	
**Years of service**				0.026
≤5 years	154	113 (73.4)	41 (26.6)	
6–10 years	105	79 (75.2)	26 (24.8)	
>10 years	60	34 (56.7)	26 (43.3)	
**Terms of contract**				0.032
Part-time	49	41 (83.7)	8 (16.3)	
Permanent	270	185 (68.5)	85 (31.5)	
**Work shift**				0.003
Day	296	216 (73.3)	80 (27.0)	
Night	23	10 (43.5)	13 (56.5)	
**Department**				0.002
Administration	104	87 (83.6)	17 (16.4)	
Production	152	97 (63.8)	55 (36.2)	
Other ^c^	63	42 (66.7)	21 (33.3)	
**Industry sector**				<0.001
Agricultural	55	22 (40.0)	36 (60.0)	
Building/construction/mining	53	44 (83.0)	9 (17.0)	
Commerce and distribution	69	49 (71.0)	20 (29.0)	
Manufacturing	142	111 (78.2)	31 (21.8)	
**Company years of operation**				<0.001
≤20 years	174	157 (90.2)	17 (9.8)	
>20 years	145	69 (47.6)	76 (52.4)	
**Number of employees**				0.192
≤25 employees	145	108 (74.5)	37 (25.5)	
>25 employees	174	118 (67.8)	56 (32.2)	
**Employees’ implementation recommendations** ^d^				0.018
Some recommendation	43	37 (86.0)	6 (14.0)	
All recommendations	276	189 (68.5)	87 (31.5)	
**Implementation barriers** ^e^				<0.001
Low	123	93 (75.6)	30 (24.4)	
Moderate	130	101 (77.7)	29 (22.3)	
High	66	32 (48.5)	34 (51.5)	

^a^, ZNOSHP knowledge had three indicators (i.e., awareness, training, and availability of ZNOSHP of policy document) categorized as low = 1 indicator, moderate = 2 indicators, and high = all 3 indicators; ^b^, Senior position include manager and supervisor while junior position includes skilled and unskilled laborers; ^c^, Other department include sales, purchasing, stores, security, and other; ^d^, All recommendations for implementation included all the six indicators (i.e., regular training to gain skills and safety knowledge, involving workers in the implementation process, empowering safety and health officers, penalties for non-compliance, familiarizing with the key policy requirements, and raising awareness on ZNOSHP by policy makers), otherwise some had ≤5 indicators; ^e^, Implementation barriers had seven indicators (i.e., lack of skills and knowledge, lack of management commitment and vision, inadequate knowledge on policy benefits, high implementation cost, disempowered safety and health professionals, lack of penalties for non-implementation, and unrealistic safety policy) categorized as low = 0–2 barriers, moderate = 3–4 barriers, and high = 5–7 barriers; ZNOSHP, Zimbabwe National Occupational Safety and Health Policy; ^†^, Chi-Square was used to test the differences in the categorical indicators. bold: distinguish the variable name from the categories.

**Table 3:** Negative binomial regression of the implementation of the ZNOHSP in Willowvale industrial area, Harare, *n* = 309.

	Implementation of ZNOSHP, IRR (95% CI)	
	Crude Model	Adjusted Model
**Employee ZNOSHP Knowledge** ^a^		
Low	1	1
Moderate	1.04 (0.93, 1.15)	1.02 (0.93, 1.12)
High	1.44 (1.28, 1.61) ****	1.32 (1.19, 1.46) ****
**Gender**		
Male	1	1
Female	1.01 (0.91, 1.13)	0.95 (0.87, 1.04)
**Age group**		
≤29	1	1
30–39	1.01 (0.89, 1.13)	0.97 (0.87, 1.07)
≥40	1.06 (0.92, 1.21)	0.99 (0.88, 1.12)
**Education level**		
Secondary and below	1	1
College/university	0.94 (0.85, 1.04)	0.99 (0.91, 1.08)
**Job position** ^b^		
Senior	1	1
Junior	1.05 (0.95, 1.15)	1.02 (0.94, 1.11)
**Years of service**		
≤5 years	1	1
6–10 years	0.98 (0.88, 1.09)	0.95 (0.87, 1.04)
>10 years	1.18 (1.04, 1.34) ***	1.05 (0.94, 1.18)
**Terms of contract**		
Part-time	1	1
Permanent	1.17 (1.02, 1.34) **	1.04 (0.92, 1.17)
**Work shift**		
Day	1	1
Night	1.34 (1.13, 1.59) ****	1.12 (0.96, 1.30)
**Department**		
Administration	1	1
Production	1.25 (1.13, 1.39) ****	1.13 (1.03, 1.26) **
Other ^c^	1.23 (1.07, 1.40) ***	1.11 (0.99, 1.25) *
**Industry sector**		
Agricultural	1	1
Building/construction/mining	0.70 (0.61, 0.82) ****	1.02 (0.88, 1.19)
Commerce and distribution	0.77 (0.67, 0.89) ****	0.98 (0.85, 1.12)
Manufacturing	0.69 (0.62, 0.79) ****	0.90 (0.80, 1.02) *
**Company years of operation**		
≤20 years	1	1
>20 years	1.49 (1.37, 1.62) ****	1.33 (1.21, 1.46) ****
**Number of employees**		
≤25 employees	1	1
>25 employees	1.09 (0.99, 1.20) *	0.99 (0.91, 1.08)
**Employees’ implementation recommendations** ^d^		
Some recommendations	1	1
All recommendations	1.18 (1.03, 1.36) **	1.05 (0.93, 1.18)
**Implementation barriers** ^e^		
Low	1	1
Moderate	0.94 (0.85, 1.05)	0.96 (0.88, 1.06)
High	1.28 (1.13, 1.44) ****	1.12 (1.01, 1.25) **

^a^, ZNOSHP knowledge had three indicators (i.e., awareness, training, and availability of ZNOSHP of policy document) categorized as low = 1 indicator, moderate = 2 indicators, and high = all 3 indicators; ^b^, Senior position include manager and supervisor while junior position includes skilled and unskilled laborers; ^c^, Other department include sales, purchasing, stores, security, and other; ^d^, All recommendations for implementation included all the six indicators (i.e., regular training to gain skills and safety knowledge, involving workers in the implementation process, empowering safety and health officers, penalties for non-compliance, familiarizing with the key policy requirements, and raising awareness on ZNOSHP by policy makers), otherwise some had ≤5 indicators; ^e^, Implementation barriers had seven indicators (i.e., lack of skills and knowledge, lack of management commitment and vision, inadequate knowledge on policy benefits, high implementation cost, disempowered safety and health professionals, lack of penalties for non-implementation, and unrealistic safety policy) categorized as low = 0–2 barriers, moderate = 3–4 barriers, and high = 5–7 barriers; ZNOSHP, Zimbabwe National Occupational Safety and Health Policy; IRR, incidence rate ratio; CI, confidence interval; * *p* ≤ 0.10; ** *p* ≤ 0.05; *** *p* ≤ 0.01; **** *p* ≤ 0.001. bold: distinguish the variable name from the categories.

## Data Availability

We have yet not publicly reported any scientific data of the study. This is the final degree work by the Master of Public Health (MPH) student; nevertheless, these data are not published in any other publication.
